# Role of IQGAP3 in metastasis and epithelial–mesenchymal transition in human hepatocellular carcinoma

**DOI:** 10.1186/s12967-017-1275-8

**Published:** 2017-08-15

**Authors:** Yongjie Shi, Nan Qin, Qiang Zhou, Yanqiu Chen, Sicong Huang, Bo Chen, Gang Shen, Hongyun Jia

**Affiliations:** 1 Department of Clinical Examination, The Second Affiliated Hospital of Guangzhou Medical University, Guangzhou, 510260 Guangdong People’s Republic of China; 2 Department of ENT, Guangzhou Women and Children’s Medical Center, Guangzhou Medical University, 9th Jinsui Road, Guangzhou, 510623 Guangdong People’s Republic of China; 3 Department of Interventional Radiology and Vascular Anomalies, Guangzhou Women and Children’s Medical Center, Guangzhou Medical University, 9th Jinsui Road, Guangzhou, 510623 Guangdong People’s Republic of China

**Keywords:** IQGAP3, Hepatocellular carcinoma, Metastasis, Epithelial–mesenchymal transition, TGF-β signaling

## Abstract

**Background:**

Hepatocellular carcinoma (HCC) is one of the most lethal cancers worldwide owing to its high rates of metastasis and recurrence. The oncogene IQ motif-containing GTPase activating protein 3 (*IQGAP3*) is ubiquitously overexpressed in several human cancers, including liver, ovary, lung, large intestine, gastric, bone marrow, and breast malignancies and is involved in the invasion and metastasis of cancer cells. Therefore, we aimed to determine the biological role and molecular mechanism of IQGAP3 in HCC.

**Methods:**

We used 120 archived clinical HCC samples, 9 snap-frozen HCC tumor tissues, and 4 normal liver tissues. Expression of IQGAP3 mRNA and protein in HCC cell lines (Hep3B, SMMC-7721, HCCC-9810, HepG2, BEL-7404, HCCLM3, QGY-7701, Huh7, and MHCC97H) and normal liver epithelial cells LO2 was examined by western blot, quantitative polymerase chain reaction, and immunohistochemistry. In addition, wound-healing and transwell matrix penetration assays were used to assess the migratory and invasive abilities of HCC cells, respectively.

**Results:**

Expression of the IQGAP3 was robustly upregulated in HCC cells and tissues. High expression of IQGAP3 in HCC correlated with aggressive clinicopathological features and was an independent poor prognostic factor for overall survival. Furthermore, ectopic expression of IQGAP3 markedly enhanced HCC cell migration, invasion, and epithelial-to-mesenchymal transition (EMT) in vitro and promoted metastasis of orthotopic hepatic tumors in nude mice. Conversely, silencing endogenous *IQGAP3* showed an opposite effect. Mechanistically, IQGAP3 promoted EMT and metastasis by activating TGF-β signaling.

**Conclusions:**

IQGAP3 functions as an important regulator of metastasis and EMT by constitutively activating the TGF-β signaling pathway in HCC. Our findings present new evidence of the role of IQGAP3 in EMT and metastasis, indicating its potential as a prognostic biomarker candidate and a therapeutic target against HCC.

## Background

Human hepatocellular carcinoma (HCC), one of the most common primary malignancies of the liver, is the third-leading cause of cancer mortality worldwide and the second-leading cause of cancer mortality in China [1–3]. Although significant improvements have been made in its early diagnosis and systemic treatment in the last two decades, the prognosis of HCC remains poor because of its high rates of recurrence and metastasis [4]. Therefore, it is critical to explore the molecular mechanisms underlying the progression and metastasis of HCC, identify valuable factors to predict its prognosis, and develop novel therapeutic strategies for HCC.

A transient phenomenon involved in the metastasis of various types of cancers is epithelial-to-mesenchymal transition (EMT), which plays a key role in the invasion and metastasis of tumor cells [5]. The initiation of EMT is triggered by several EMT-inducing transcription factors [6] including Snail1/2, Twist1/2, and zinc-finger E-box binding homeobox 1/2 [7–9]. Emerging evidence suggests that EMT contributes to tumor metastasis and recurrence of various cancers including HCC. Transforming growth factor (TGF)-β is the most important inducer of EMT in these cancers, as it stimulates the expression of EMT-inducing transcription factors [10–12]. When TGF-β signaling is activated, cancer cells acquire access to the EMT program, lose their epithelial characteristics including polarity and specialized cell–cell contacts, and acquire migratory capacity, allowing them to invade the surrounding tissues, lymphatic and blood vessels, and even remote locations [13–15]. Therefore, TGF-β signaling-associated induction of the EMT is considered a key step in the progression of tumor metastasis.

IQ motif-containing GTPase activating protein 3 (IQGAP3), a primary member of the IQGAP family and GTPase-activating protein, is located at 1q21.3, which is a hotspot for gene amplification in cancer. IQGAP3 was initially found to be an effector of Rac1 and Cdc42 [16], but current data have demonstrated that it is involved in many essential cellular processes including cell proliferation, cytoskeleton remodeling, growth factor receptor signaling, and cell adhesion. In addition, it functions as an oncogene and is ubiquitously overexpressed in several human cancers including liver, ovary, lung, large intestine, gastric, bone marrow, and breast malignancies [17]. Furthermore, several lines of evidence indicate that it is involved in the invasion and metastasis of cancer cells. Yang et al. reported that IQGAP3 promotes metastasis of lung cancer cells by activating EGFR–ERK signaling [17]. Moreover, Wu et al. showed that genes regulating the cytoskeleton-remodeling processes are frequently altered, especially in metastatic lung adenocarcinoma and that high expression of IQGAP3 is a crucial marker for poor prognosis [18]. Notably, gene expression profiling has shown that IQGAP3 is upregulated in HCC [19].

Considering the importance of IQGAP3 in cancers, in this study, we aimed to determine the biological role and molecular mechanism of IQGAP3 in HCC.

## Methods

### Tissue specimens and clinicopathological characteristics

A total of 120 paraffin-embedded, archived, histopathologically and clinically diagnosed, clinical HCC samples were collected at the Second Affiliated Hospital of Guangzhou Medical University from 2007 to 2009. In addition, we collected 9 snap-frozen HCC tumor tissues and four normal liver tissues from patients who underwent surgery at the Second Affiliated Hospital of Guangzhou Medical University between 2014 and 2015. The clinicopathological characteristics of the samples are presented in Table 1.

**Table 1:** Clinicopathological characteristics of patient samples and expression of IQGAP3 in HCC

Clinical character	Variable	No. of patients (%)
Age (years)	≤50	53 (44.2)
	>50	67 (55.8)
Gender	Female	13 (10.8)
	Male	107 (89.2)
Clinical stage	I	48 (40.0)
	II	44 (36.7)
	III	21 (17.5)
	IV	7 (5.8)
Cirrhosis	No	58 (48.3)
	Yes	62 (51.7)
T classification	T1	53 (44.2)
	T2	12 (10.0)
	T3	6 (5.0)
	T4	49 (40.8)
N classification	N0	97 (80.8)
	N1	23 (19.2)
M classification	M0	113 (94.2)
	M1	7 (5.8)
HCV	No	119 (99.2)
	Yes	1 (0.8)
HBsAg	Negative	17 (14.2)
	Positive	103 (85.8)
Vital status	Alive	17 (14.2)
	Death	103 (85.8)
IQGAP3 expression	Low expression	68 (56.7)
	High expression	52 (43.3)

*IQGAP3* IQ motif-containing GTPase activating protein 3

Prior patient consent and approval from the Institutional Research Ethics Committee were obtained. All samples were used for research purposes only.

### Cell lines and cell culture

LO2, HepG2, MHCC97H, HCCLM3, and SMMC-7721 cell lines were purchased from the Institute of Chemistry and Cell biology (Shanghai, China). Hep3B, HCCC-9810, BEL-7404, QGY-7701, and Huh7 were purchased from the American Type Culture Collection (Manassas, VA). HCC cell lines including Hep3B, SMMC-7721, HCCC-9810, HepG2, BEL-7404, HCCLM3, QGY-7701, Huh7, and MHCC97H were cultured in Dulbecco’s modified Eagle’s medium (Invitrogen, Carlsbad, CA, USA) supplemented with 10% fetal bovine serum (HyClone, Logan, UT, USA). Normal liver epithelial cells—LO_2_—were maintained in a bronchial epithelial growth medium (Clonetics Corporation, Walkersville, MD), supplemented with 5 ng/mL epithelial growth factor, 70 ng/mL phosphorylethanolamine, and 10% fetal bovine serum. Cells were maintained in a humidified atmosphere at 37 °C with 5% CO_2_.

### RNA extraction and real-time quantitative polymerase chain reaction (qPCR)

Total RNA from cultured cells and fresh surgical HCC tissues was extracted using the Trizol reagent (Invitrogen), and 2 μg of RNA from each sample was used for cDNA synthesis. Real-time PCR was performed using FastStart Universal SYBR Green Master (ROX; Roche, Basel, Switzerland) on a 7500 Real-Time PCR system (Applied Biosystems). The primers used for qRT-PCR were as follows: IQGAP3, 5′-AGGGTGATCAGGAACAAGCC-3′ (forward) and 5′-ACAGGGTACACTGGAGGCAG-3′ (reverse); TGF-β1, 5′-GGCCCTGCCCCTACATTT-3′ (forward) and 5′-CCGGGTTATGCTGGTTGTACA-3′ (reverse); matrix metalloproteinase-2 (MMP2), 5′-GGAAAGCCAGGATCCATTTT-3′ (forward) and 5′-ATGCCGCCTTTAACTGGAG-3′ (reverse); thrombospondin 1 (THBS1), 5′-CACAGCTCGTAGAACAGGAGG-3′ (forward) and 5′-CAATGCCACAGTTCCTGATG-3′ (reverse); latent transforming growth factor beta binding protein-1 (LTBP1), 5′-CTTGGGCTTGAGCACGTATT-3′ (forward) and 5′-GCCCAGATGACCTTAACCCT-3′ (reverse); and glyceraldehyde 3-phosphate dehydrogenase (GAPDH), 5′-TTGAGGTCAATGAAGGGGTC-3′ (forward) and 5′-GAAGGTGAAGGTCGGAGTCA-3′ (reverse). Conditions for the PCR reactions were as follows: 10 min at 95 °C followed by 40 cycles of 15 s at 95 °C and 1 min at 60 °C. Relative expression levels were calculated as 2^−(ΔΔCt)^.

To investigate the clinical significance and biological role of IQGAP3 in HCC, we first analyzed the mRNA expression of IQGAP3 in HCC tissues using published data from The Cancer Genome Atlas (TCGA). We analyzed publicly available gene expression array data for liver cancers using Gene Set Enrichment Analysis (GSEA). Data were acquired from the TCGA data portal https://portal.gdc.cancer.gov/projects/TCGA-LIHC.

### Western blot analysis

Western blot was performed as previously described [20], using anti-IQGAP3 (ab118258, Abcam), anti-E-cadherin, anti-N-catenin, anti-fibronectin, anti-vimentin, anti-p-Smad2, anti-p-Smad3, anti-Smad2, and anti-Smad3 antibodies (Cell Signaling, Danvers, MA, USA). The membranes were stripped and re-probed with anti-GAPDH (Proteinch, Chicago, USA) as a loading control.

### Immunohistochemistry (IHC)

IHC analysis was performed to examine IQGAP3 expression in 120 human HCC specimens, as previously described [21]. The degree of immunostaining of formalin-fixed, paraffin-embedded sections was reviewed and scored by two independent observers who were blinded to the histopathological features and patient data of the samples. IQGAP3 expression was evaluated according to the proportion of positively stained tumor cells and intensity of staining. Tumor cell proportions were scored as follows: 0, no positive tumor cells; 1, <10% positive tumor cells; 2, 10–35% positive tumor cells; 3, 35–75% positive tumor cells; and 4, >75% positive tumor cells. The staining intensity of protein expression was graded according to the following criteria: 1, no staining; 2, weak staining (light yellow); 3, moderate staining (yellow brown); and 4, strong staining (brown).

The staining-intensity score and percentage of staining were then multiplied to yield an IQGAP3 staining index (SI), with possible scores of 0, 2, 3, 4, 6, 8, 9, 12, and 16. Samples with an SI ≥ 8 were considered to have high expression and samples with an SI < 8 were considered to have low expression. Cutoff values for IQGAP3 were determined on the basis of a measure of heterogeneity by using the log-rank test with respect to overall survival.

### Vectors, retroviral infections, and inhibitors

Human IQGAP3 cDNA was PCR-amplified and cloned into a pSin-EF2 vector (Clontech, Mountain View, CA, USA). To silence endogenous *IQGAP3*, 2 short hairpin RNAs (shRNA) against IQGAP3 in pLKO-puro vector were purchased (Sigma-Aldrich). Luciferase cDNA was PCR-amplified and cloned into the pMSCV-neo-retro vector (Clontech). Stable cell lines expressing IQGAP3 or IQGAP3 shRNA were selected for 10 days with 0.5 μg/mL puromycin at 48 h after infection. The shRNA sequences were as follows: IQGAP3-RNAi#1: CCGGCCTCGCCATGACTGATAAGTTCTCGAGAACTTATCAGTCATGGCGAGGTTTTTG, IQGAP3-RNAi#2: CCGGGCCAAAGTCAATGTCAACCTTCTCG.

AGAAGGTTGACATTGACTTTGGCTTTTTG, and Smad3-RNAi: CTGTCCAATGTCAACCGGAAT. The TGF-β inhibitor SB431542 was purchased from Selleckchem (Houston, TX, USA) and dissolved in dimethyl sulfoxide.

### Wound-healing assay

Cell migratory ability was assessed using the wound scratch assay. Briefly, cells were seeded into 6-well plates containing 1 × 10^6^ cells/well. A scratch was made using a 10-μL sterile pipette tip in a confluent cell monolayer. Images were captured on an inverted Olympus IX50 microscope at 0, 24, and 36 h after wounding. Eight images per treatment were analyzed to determine the average position of the migrating cells at the edges of the wounds. All experiments were repeated three times.

### Transwell matrix invasion assay

The transwell matrix assay was used to assess the invasiveness of HCC cell. Cells (4 × 10^4^) were plated into the upper chamber of polycarbonate transwell filters coated with Matrigel (BD Biosciences, San Jose, CA) and cultured at 37 °C for 24 h. Thereafter, the cells inside the upper chamber were removed with cotton swabs, and cells that had migrated to the bottom surface of the membrane were fixed in 1% paraformaldehyde, stained with hematoxylin, and counted in 10 random fields of view per well.

### Three-dimensional spheroid invasion assay

Cells (1 × 10^4^) were trypsinized and seeded in 24-well plates coated with 2% Matrigel (BD Biosciences). The medium was refreshed every alternate day, and images of the cells were taken using a light microscope at 2-day intervals for 2 weeks.

### Immunofluorescence analysis

Stably transfected cells were seeded in 24-well culture plates (Corning Costar Corp, Corning, NY, USA) to prepare for immunofluorescence analysis and incubated with primary antibodies against E-cadherin and vimentin. The cells were subsequently incubated with rhodamine-conjugated goat antibodies against rabbit or mouse IgG (Jackson Immuno Research Laboratories, West Grove, PA). The cover slips were counterstained with 4′-6-diamidino-2-phenylindole and imaged with a confocal laser-scanning microscope (Olympus FV1000). Data were processed using Adobe Photoshop 7.0 software.

### Animal experiments

An orthotopic hepatic tumor model in nude mice (3–4 weeks of age, male, BALB/c) was established. Briefly, approximately 1 × 10^6^ cells in a 25 μL culture medium (cells:matrigel, 1:1.5) were injected subcutaneously under the liver capsule of the mice (6 animals in each group). The survival status of nude mice was recorded every week. After 6 weeks, the animals were killed by cervical dislocation and autopsied. The length (L) and width (W) of the tumors were measured during the autopsy, and the volume (V) was calculated as V = ½ (L × W^2^). Intrahepatic metastasis was defined as the presence of nodules at a distance from the in situ tumors. The livers were dissected and prepared for hematoxylin and eosin (H & E) staining. Expression of IQGAP3, Snail, Twist1, MMP2, MMP9, and EMT markers in tumors was examined using western blot.

### Statistical analysis

All statistical analyses were performed using SPSS19.0 statistical software package. Groups were compared using the Chi square test. Univariate and multivariate survival analyses were performed using Cox regression analysis. Survival curves were plotted using the Kaplan–Meier method and compared by the log-rank test. In all cases, a *P* < 0.05 was considered statistically significant.

## Results

### IQGAP3 is upregulated in human HCC cell lines and tissues

**Fig. 1:**
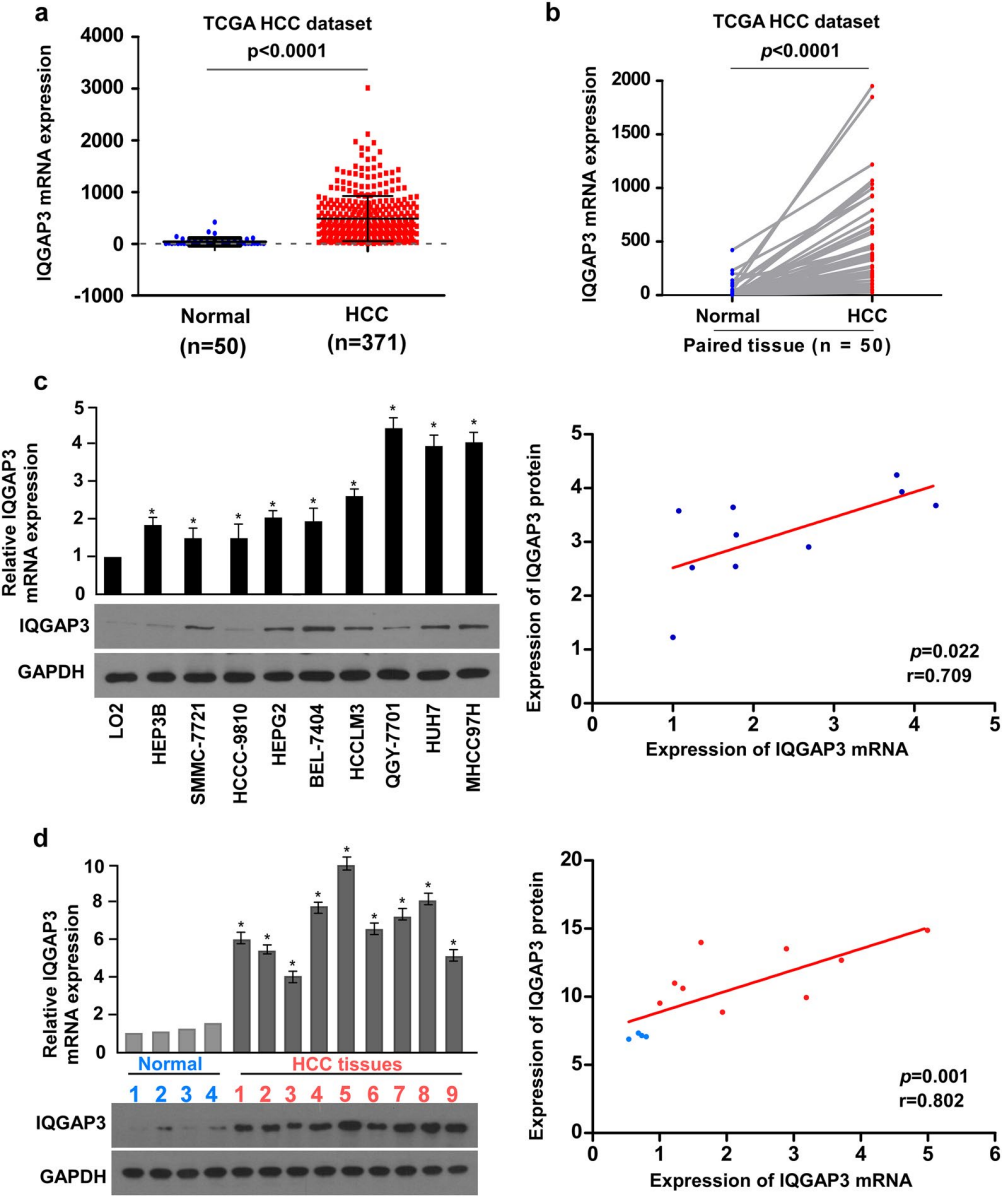
IQGAP3 expression is upregulated in HCC. IQGAP3 mRNA levels in liver cancer tissues are assessed by analyzing The Cancer Genome Atlas liver cancer mRNA data set in **a** normal (n = 50) and HCC (n = 371) tissues and **b** the 50 paired adjacent non-tumor tissues (NT) and HCC tissues (T). *Lines* represent mean ± SD. P < 0.0001, *t* test. **c** Expression of IQGAP3 mRNA and protein in HCC cell lines (Hep3B, SMMC-7721, HCCC-9810, HepG2, BEL-7404, HCCLM3, QGY-7701, Huh7, and MHCC97H) and normal liver epithelial cells LO_2_, as examined by western blotting and quantitative polymerase chain reaction. **d** Western blot and real-time polymerase chain reaction analyses of IQGAP3 expression in 4 normal liver tissues and 9 liver tumor tissues from HCC patients. Glyceraldehyde 3-phosphate dehydrogenase is used as a loading control (*left panel*). Correlation analysis is used for IQGAP3 mRNA and protein expression (*right panel*). *Each bar* represents the mean ± SD of three independent experiments. *P < 0.05. *HCC* hepatocellular carcinoma, *IQGAP3* IQ motif-containing GTPase activating protein 3, *SD* standard deviation

Analysis of the mRNA expression of IQGAP3 in HCC tissues showed that IQGAP3 levels remained low in non-tumor liver tissues, but increased significantly in patients with HCC (P < 0.0001), suggesting that IQGAP3 might contribute to the high cell-proliferation rates in HCC (Fig. 1a). In addition, TCGA data analysis revealed that IQGAP3 levels were significantly upregulated in liver cancer tissues as compared to paired tumor-adjacent non-tumor tissues (Fig. 1b). Furthermore, we verified IQGAP3 expression in liver cancer cell lines and fresh tissues. Real-time PCR and western blotting revealed that IQGAP3, at both the mRNA and protein levels, was markedly overexpressed in all 9 tested liver cancer cell lines as compared to the immortalized normal liver epithelial cells (Fig. 1c). Similarly, the mRNA and protein levels of IQGAP3 were differentially upregulated in all 9 freshly frozen liver cancer samples as compared to the 4 non-tumor tissues (Fig. 1d), suggesting that IQGAP3 is upregulated in liver cancer cell lines and liver cancer tissues.

### Upregulation of IQGAP3 expression is correlated with poor prognosis and metastasis in HCC

**Fig. 2:**
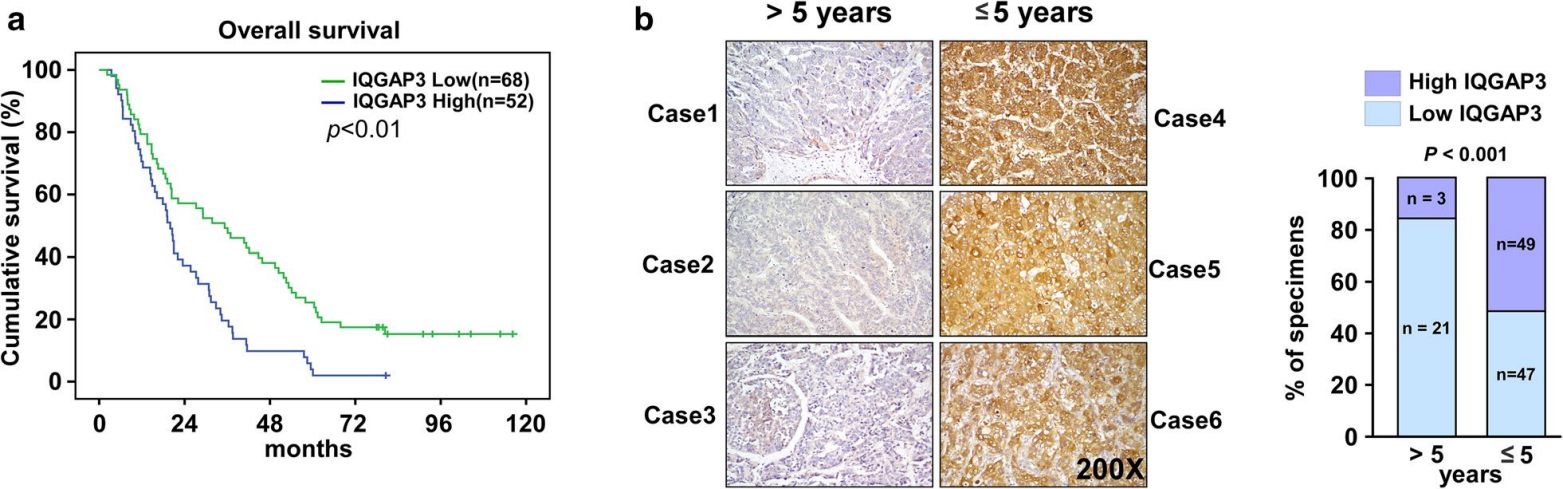
Upregulation of IQGAP3 is correlated with poor prognosis in human HCC. **a** Kaplan–Meier overall survival curves for HCC patients with low and high levels of IQGAP3 expression (n = 120; log-rank test, *p* < 0.01). **b** Representative immunohistochemistry analyses with high and low levels of IQGAP3 expression in tumor tissues from patients with HCC (>5 years, n = 24 and ≤5 years, n = 96). *p* < 0.001. *HCC* hepatocellular carcinoma, *IQGAP3* IQ motif-containing GTPase activating protein 3

IQGAP3 expression of 120 paraffin-embedded HCC tissues was examined by IHC. IQGAP3 expression was closely associated with poor prognosis and metastasis. Kaplan–Meier and long-rank tests for survival analysis revealed that patients with high IQGAP3 levels had a significantly poorer overall survival than patients with low IQGAP3 levels (Fig. 2a). Furthermore, patients with an overall survival time of <5 years had higher IQGAP3 expression than patients with a survival of >5 years (Fig. 2b). In addition, the Chi square test and Spearman correlation analysis revealed that the IQGAP3 levels were significantly correlated with clinical stage, N classification, M classification, and vital status in HCC patients (all P < 0.05; Tables 2, 3), indicating that IQGAP3 may be correlated with HCC metastasis. Multivariate analyses revealed that IQGAP3 expression was recognized as an independent prognostic factor in HCC (P < 0.05; Table 4), suggesting that IQGAP3 has potential clinical value as a predictive biomarker for disease outcome in HCC.

**Table 2:** Correlation between IQGAP3 expression and clinicopathologic characteristics of HCC

Clinical character	Variable	Total	IQGAP3		Chi square test, *P* value	Fisher’s exact test, *P* value
			Low-expression, no. cases %	High-expression, no. cases %		
Age (years)	≤50	53	31 (25.8)	22 (18.3)	0.720	0.853
	>50	67	37 (30.8)	30 (25.0)		
Gender	Female	13	5 (4.2)	8 (6.7)	0.161	0.236
	Male	107	63 (52.5)	44 (36.7)		
Clinical stage	I	48	31 (25.8)	17 (14.2)	0.001	
	II	44	31 (25.8)	13 (10.8)		
	III	21	6 (5.0)	15 (12.5)		
	IV	7	0 (0.0)	7 (5.8)		
Cirrhosis	No	58	32 (26.7)	26 (21.7)	0.749	0.854
	Yes	62	36 (30.0)	26 (21.7)		
T classification	T1	53	27 (22.5)	26 (21.7)	0.460	
	T2	12	7 (5.8)	5 (4.2)		
	T3	6	5 (4.2)	1 (0.8)		
	T4	49	29 (24.2)	20 (16.7)		
N classification	N0	97	62 (51.7)	35 (29.2)	0.001	0.002
	N1	23	6 (5.0)	17 (14.2)		
M classification	M0	113	68 (56.7)	45 (37.5)	0.002	0.002
	M1	7	0 (0.0)	7 (5.8)		
HCV	No	119	67 (55.8)	52 (43.3)	0.380	1.000
	Yes	1	1 (0.8)	0 (0.0)		
HBsAg	Negative	17	8 (6.7)	9 (7.5)	0.388	0.436
	Positive	103	60 (50.0)	43 (35.8)		
Vital status	Alive	17	15 (12.5)	2 (1.7)	0.005	0.007
	Death	103	53 (44.2)	50 (41.7)		

*IQGAP3* IQ motif-containing GTPase activating protein 3

**Table 3:** Spearman correlation analysis between IQGAP3 expression and clinical pathologic factors

Variable	IQGAP3 expression	
	Spearman correlation	*P* value
Clinical stage	0.332	0.001
N classification	0.300	0.001
M classification	0.285	0.002
Vital status	0.259	0.004

*IQGAP3* IQ motif-containing GTPase activating protein 3

**Table 4:** Multivariable analyses of various prognostic parameters in patients with HCC

Variable	B	Wald	*P*	Relative risk	95% confidence interval
Age (years)	0.363	2.970	0.085	1.437	0.951–2.170
Gender	0.159	0.223	0.636	1.173	0.606–2.271
Clinical stage	0.071	0.054	0.816	1.074	0.590–1.954
Cirrhosis	−0.288	1.880	0.170	0.750	0.497–1.132
T classification	0.038	0.123	0.726	1.039	0.839–1.287
N classification	−0.416	0.674	0.412	0.660	0.244–1.781
M classification	−0.934	2.133	0.144	0.393	0.112–1.377
HCV	−11.647	0.001	0.970	0.000	0–3.964E + 259
HBsAg	−0.310	0.012	0.912	0.969	0.553–1.697
IQGAP3	0.922	15.835	0.001	2.513	1.596–3.957

### IQGAP3 modulates the growth and prognosis of HCC in vivo

**Fig. 3:**
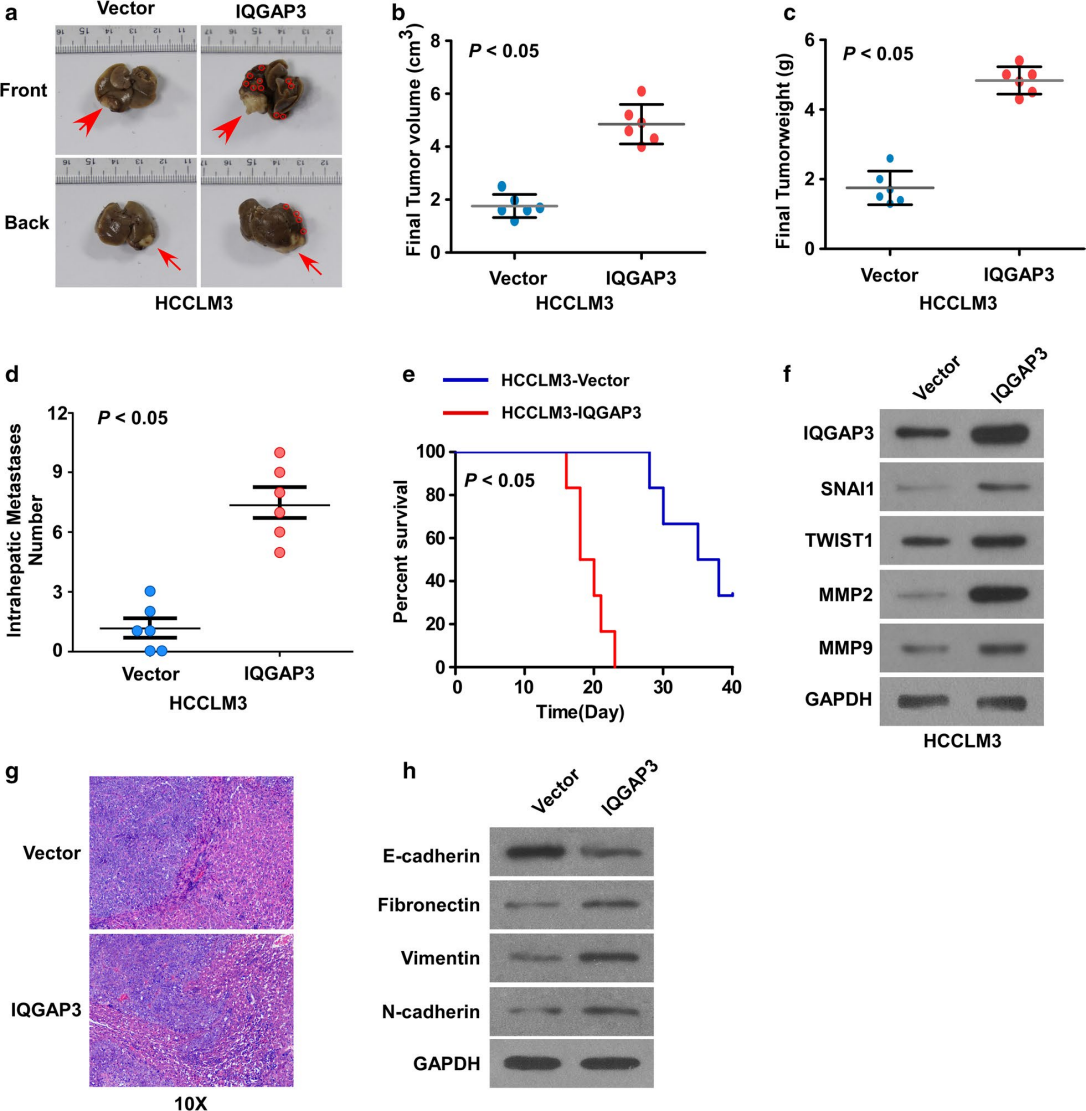
IQGAP3 modulates the growth and prognosis of HCC in vivo. **a** Representative images of tumors from each experimental group. Orthotopic hepatic tumors model in nude mice were constructed using HCCLM3 cells with IQGAP3 overexpression (HCCLM3–IQGAP3) or vector control (HCCLM3-Vector). **b** Volumes of in situ tumors in the IQGAP3-overexpressing and control groups were measured on indicated days. Data are presented as mean ± SD. **c** Tumor weights of each group. **d** IQGAP3 overexpression significantly increased the number of intrahepatic metastatic nodules as compared with the control group. Summary data of each group are shown (n = 6/group). **e** Kaplan–Meier overall survival curves show a short survival time in the overexpression group. **f** Western blot assay detects IQGAP3, Snail, Twist1, MMP2, and MMP9 expression in the indicated tumors. Scale bars, 20 μm. **g** Histological analyses of liver tumors by hematoxylin and eosin (H & E) staining. Representative images of H & E staining of liver tissue samples from different experimental groups (n = 6/group). **h** Western blot assay detects EMT marker expression in the indicated tumors. Scale bars, 20 μm Data are expressed as mean ± SD. *HCC* hepatocellular carcinoma, *IQGAP3* IQ motif-containing GTPase activating protein 3, *SD* standard deviation

To assess whether IQGAP3 affects cancer growth and prognosis in vivo, we investigated the orthotopic HCCLM3 hepatic tumors in nude mice. The IQGAP3-overexpressing tumors grew at a much higher rate in terms of size, volume, and weight than the control tumors (Fig. 3a–c). As compared with the HCCLM3-Vector group, the HCCLM3–IQGAP3 group showed a dramatic increase in the intrahepatic metastasis nodules (P < 0.05; Fig. 3a, d). Moreover, mice injected with HCCLM3–IQGAP3 showed shorter survival time than those in the control group (P < 0.05; Fig. 3e). Expression levels of IQGAP3 in hepatic tumors were further examined by western blot. IQGAP3 was robustly upregulated in tumors formed by HCCLM3/IQGAP3 cells than by the vector cells. In addition, the HCCLM3–IQGAP3 group displayed higher Snail, Twist1, MMP2, and MMP9 expression (Fig. 3f). Moreover, representative H & E staining (Fig. 3g) of liver tissues obtained from mice confirmed the results of liver tumors. Furthermore, the expression of E-cadherin was lower in the HCCLM3–IQGAP3 group than in the control group, while the expression of Fibronectin, Vimentin and N-cadherin were higher in the HCCLM3–IQGAP3 group (Fig. 3h). Taken together, these results suggest that IQGAP3 promotes the growth and prognosis of HCC in vivo.

### IQGAP3 enhances the migration and invasion of HCC cells

**Fig. 4:**
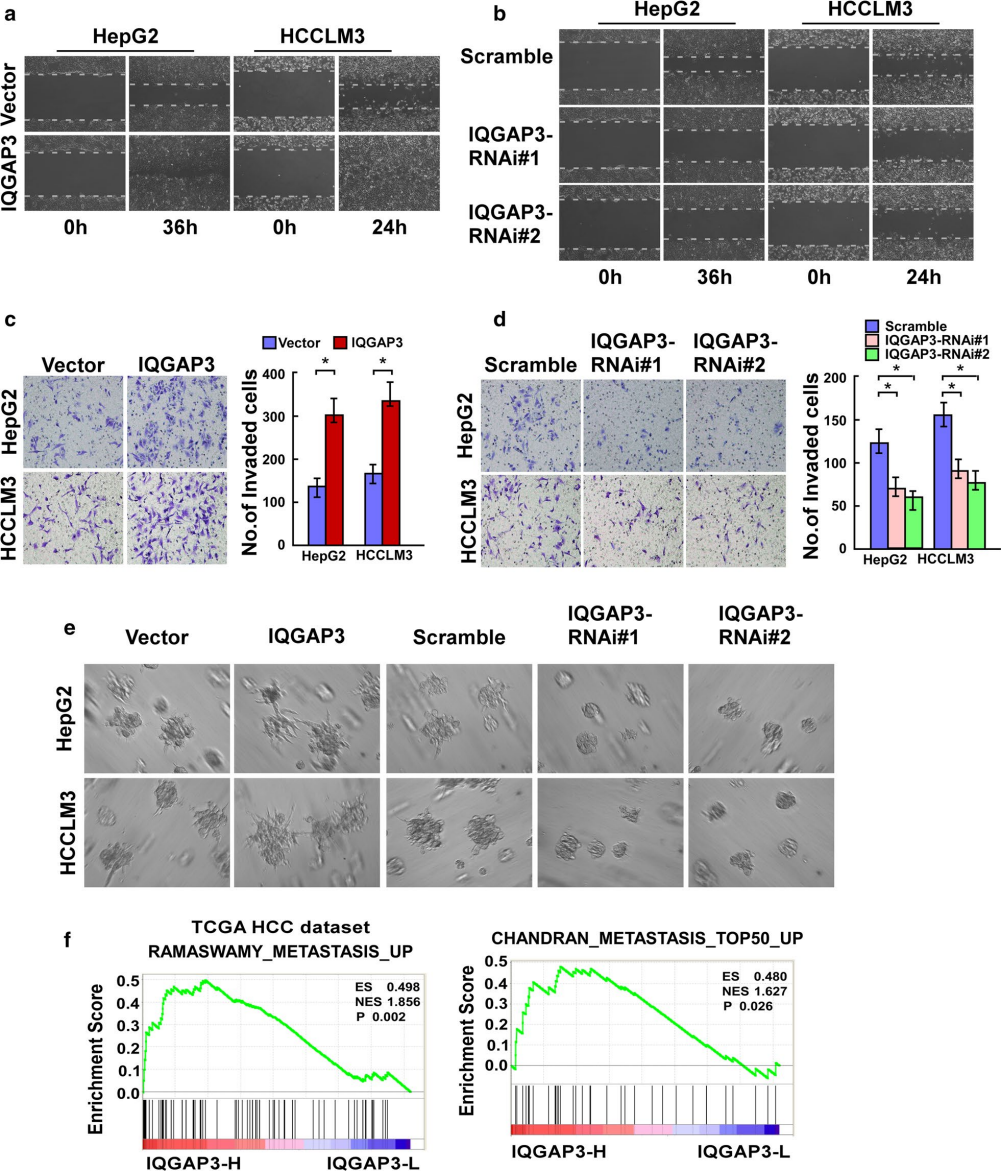
IQGAP3 enhances the migration and invasion of HCC cells. **a**, **b** Representative micrographs of the wound-healing assay in HCC cells showing the motilities of IQGAP3-overexpressing cells and IQGAP3-silenced cells at 0 and 36 h (HepG2 cell) or 24 h (HCCLM3) compared with vector controls. **c**, **d** Representative micrographs of the invasiveness of IQGAP3-overexpressing cells and IQGAP3-silenced cells compared with vector control cells in the transwell matrix penetration assay. Error bars represent mean ± SD from three independent experiments, *P < 0.05. **e** Representative micrographs of IQGAP3-transduced and IQGAP3-silenced cells cultured in the 3D spheroid invasion assay. **f** Gene Set Enrichment Analysis results indicate that IQGAP3 expression is significantly correlated with the metastasis-associated gene signatures based on The Cancer Genome Atlas HCC mRNA data set. *HCC* hepatocellular carcinoma, *IQGAP3* IQ motif-containing GTPase activating protein 3, *SD* standard deviation

To determine the function of IQGAP3 in HCC invasion and metastasis, we overexpressed and knocked down *IQGAP3* in HepG2 and HCCLM3 cells. The wound-healing and transwell matrix penetration assays revealed that overexpression of IQGAP3 significantly enhanced the migratory and invasive abilities of HCC cells compared with their respective control cells (Fig. 4a, c). Moreover, a 3D spheroid invasion assay, which is considered a good simulation of tumor invasion in vivo, revealed that IQGAP3-overexpressing cells exhibited active invasive behaviors, characterized by the formation of outward projections from individual cells (Fig. 4e). On the contrary, downregulation of IQGAP3 significantly reduced the migration and invasion of both HepG2 and HCCLM3cell lines in the wound-healing assay, transwell matrix penetration assay, and 3D spheroid formation assay (Fig. 4b, d, e). These results indicate that overexpression of IQGAP3 enhances the migration and invasion of HCC cells.

### IQGAP3 induces EMT in HCC

**Fig. 5:**
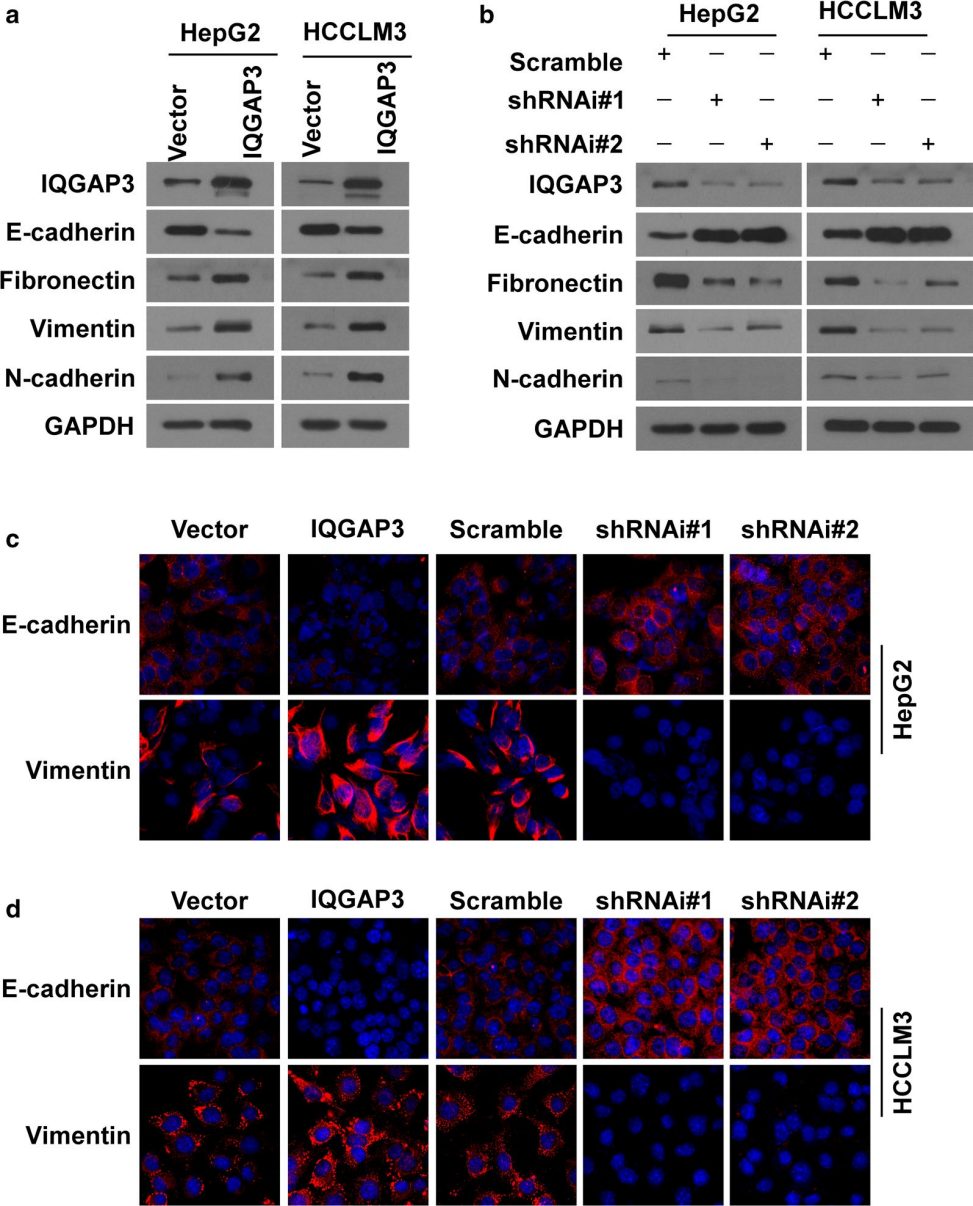
IQGAP3 induces EMT in HCC. **a**, **b** Western blot analysis of IQGAP3 and EMT marker expression in HCC cells with IQGAP3 overexpression or knockdown, and their vector control cells. **c**, **d** The immunofluorescence assay shows the relative expression of E-cadherin and vimentin in HCC cells with IQGAP3 overexpression or knockdown and their vector control cells. *HCC* hepatocellular carcinoma, *EMT* epithelial to mesenchymal transition, *IQGAP3* IQ motif-containing GTPase activating protein 3

EMT has been identified as a key step in initiating migration and invasion of cancer cells [22]. To determine whether IQGAP3 promotes invasiveness of HCC through EMT, we measured EMT biomarkers using western blot and immunofluorescence assays. Consistently, we found that HCC cells transfected with IQGAP3 expressed high IQGAP3 levels and the typical EMT phenotype, including a decrease in the expression of epithelial marker E-cadherin and an increase in expression of mesenchymal markers fibronectin, vimentin, and N-cadherin (Fig. 5a). However, silencing endogenous *IQGAP3* in HepG2 and HCCLM3 cells upregulated the expression of epithelial markers and concomitantly downregulated expression of mesenchymal markers (Fig. 5b). The EMT phenotype was confirmed by immunofluorescence in HepG2 and HCCLM3 cells (Fig. 5c, d). These results suggest that IQGAP3 is associated with EMT in HCC cells.

### IQGAP3 activates the TGF-β signaling pathway

**Fig. 6:**
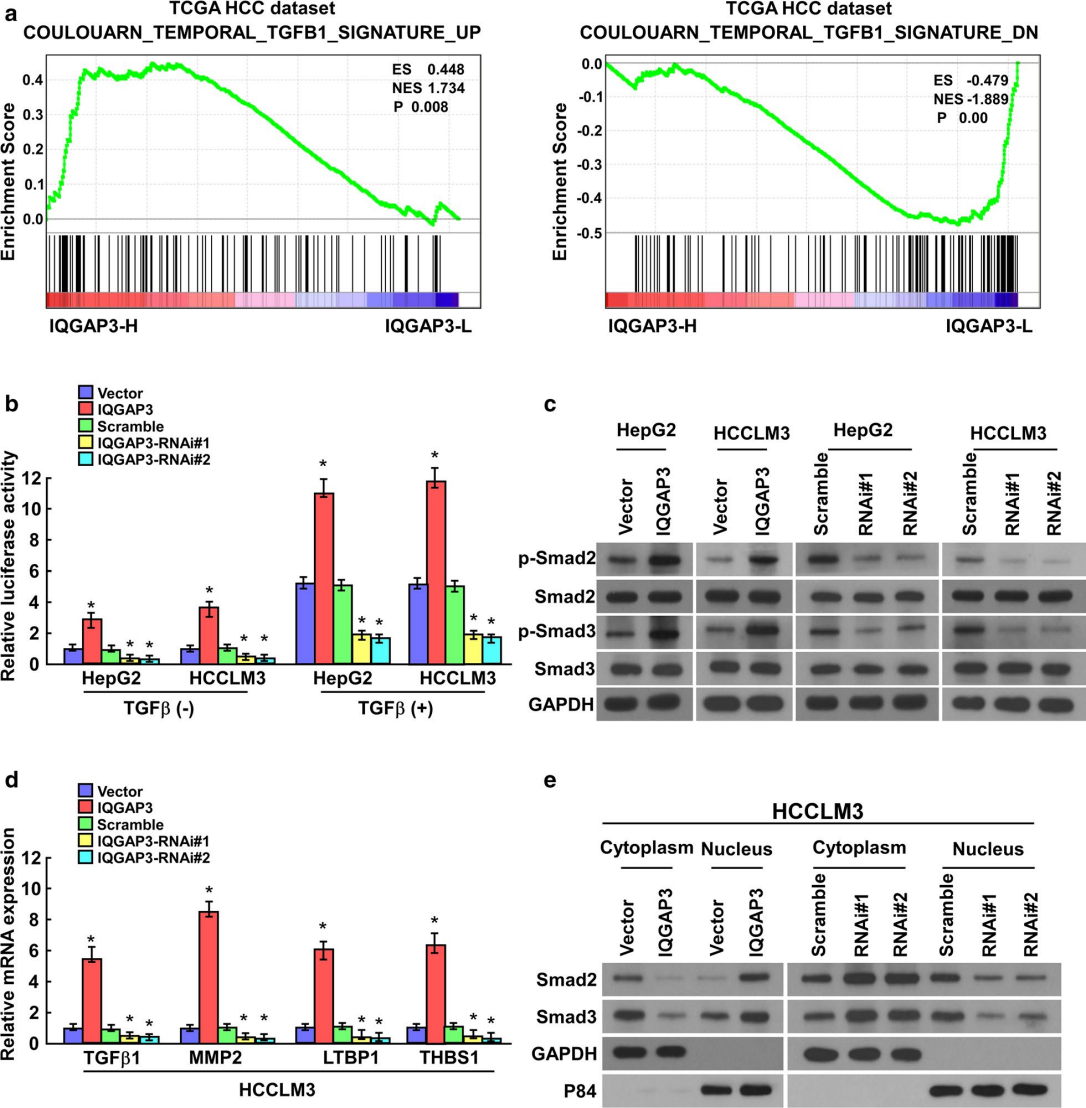
IQGAP3 activates the TGF-β signaling pathway. **a** Gene Set Enrichment Analysis shows that IQGAP3 expression is positively correlated with TGF-β–activated gene signatures (COULOUARN_TEMPORAL_TGFB1_SIGNATURE_UP and COULOUARN_TEMPORAL_TGFB1_SIGNATURE_DN). **b** TGF-β–responsive luciferase activity is measured in the indicated cells after 48 h culture with or without TGF-β1 (5 ng/mL) for 20 h using the dual luciferase assay. Values are presented as mean ± SD of triplicate samples. * *p* < 0.05. **c** Western blot analysis of the phosphorylated and total levels of Smad2 and Smad3 in the indicated HepG2 and HCCLM3 cells. GAPDH is used as a loading control. **d** IQGAP3 regulates the expression levels of numerous well-known genes downstream of TGF-β, as shown by real-time polymerase chain reaction analysis. * *p* < 0.05. **e** Western blot analysis of Smad2 and Smad3 expression in the cytoplasm and nucleus of HCCLM3 cells. GAPDH and p84 are used as loading controls for the cytoplasmic and nuclear fractions, respectively. *HCC* hepatocellular carcinoma, *IQGAP3* IQ motif-containing GTPase activating protein 3, *SD* standard deviation, *GAPDH* glyceraldehyde 3-phosphate dehydrogenase, *TGF-β* transforming growth factor-β

To further investigate the mechanism by which IQGAP3 promotes migration and invasion via EMT in HCC, we analyzed gene expression array data for liver cancers using GSEA. We found that IQGAP3 expression was positively correlated with activation of the TGF-β signaling pathway (Fig. 6a). Because TGF-β1 signaling is one of the major driving forces of EMT in HCC, we hypothesized that IQGAP3 may activate the TGF-β signaling pathway and thus enhance migration and invasion in HCC. As shown in Fig. 6b–d, we found that overexpression of IQGAP3 enhanced the TGF-β–responsive luciferase activity, levels of phosphorylated Smad2 and Smad3, and the expression of numerous well-characterized downstream genes of TGF-β signaling, but silencing of *IQGAP3* reduced these effects. Furthermore, we examined the effect of IQGAP3 on the subcellular localization of TGF-β in HCC cells. Notably, overexpression of IQGAP3 induced TGF-β translocation into the nucleus, whereas knockdown of *IQGAP3* impaired this translocation, as determined by immunoblotting of nuclear and cytoplasmic cellular fractions (Fig. 6e). Taken together, these results suggest that IQGAP3 activates TGF-β signaling pathway in HCC cells.

### TGF-β signaling activation is required for IQGAP3-induced metastasis in HCC

**Fig. 7:**
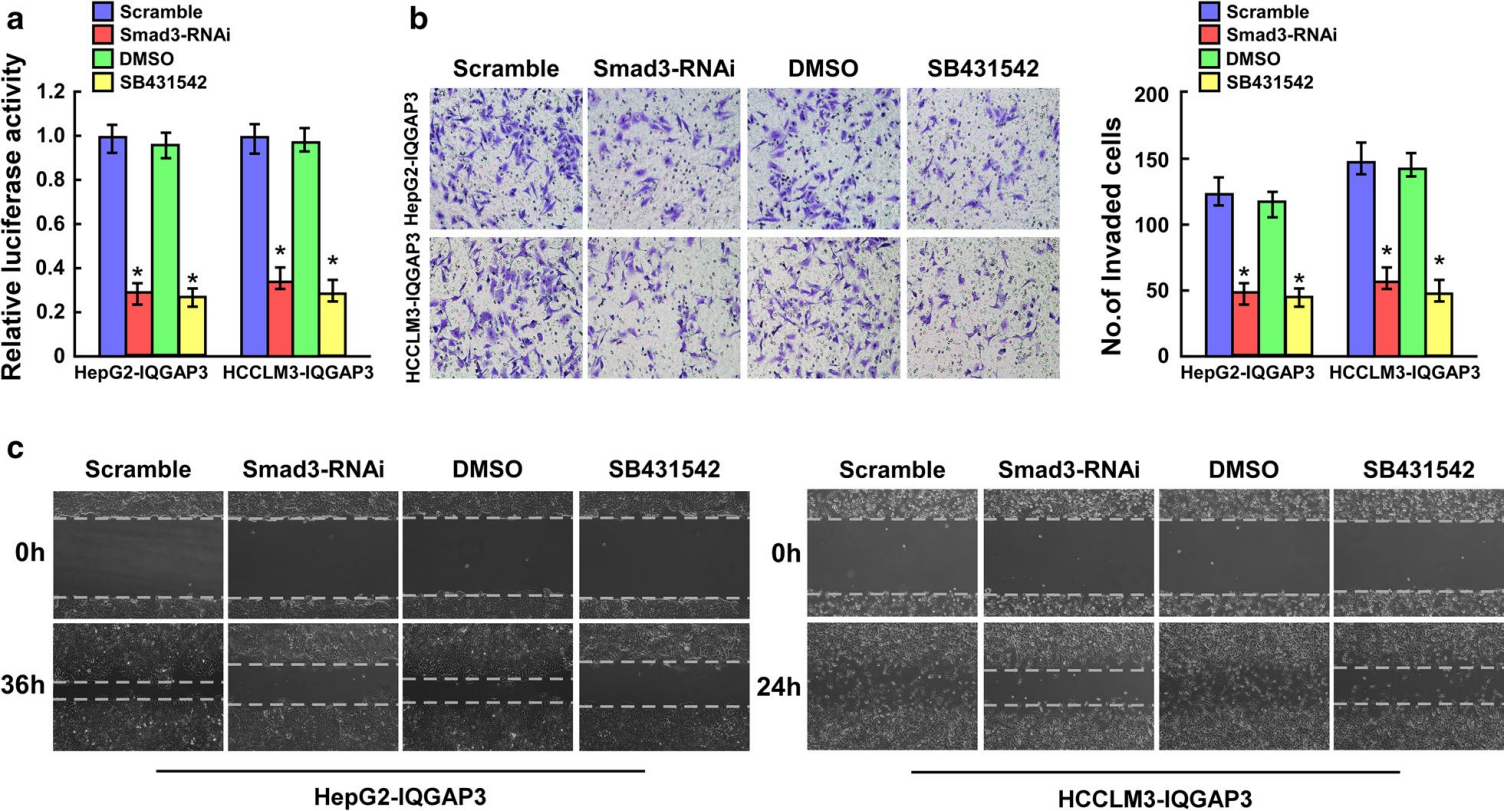
TGF-β signaling activation is required for IQGAP3-induced metastasis in HCC. **a** TGF-β–responsive luciferase activity induced by IQGAP3 is suppressed by depletion of Smad3 or treatment with TGF-β inhibitor SB431542 (10 μM) for 24 h in the indicated cells. Promotion of **b** invasion and **c** migration of HCC cells induced by IQGAP3 is inhibited by Smad3 silencing or SB431542 treatment (10 μM) for 24 h, as indicated by wound-healing and transwell matrix penetration assays. Error bars represent mean ± SD; *P < 0.05. *HCC* hepatocellular carcinoma, *IQGAP3* IQ motif-containing GTPase activating protein 3, *SD* standard deviation, *TGF-β* transforming growth factor-β

We assessed the functional significance of TGF-β signaling activation in IQGAP3-induced HCC cell metastasis by blocking TGF-β signaling in IQGAP3-overexpressing cells by silencing *Smad3* or treating cells with the TGF-β inhibitor SB431542. As expected, the stimulatory effect of IQGAP3 on TGF-β signaling activation was inhibited by silencing *Smad3* or SB431542 treatment (Fig. 7a). Moreover, silencing *Smad3* and SB431542 treatment both abrogated the effects of IQGAP3 on HCC cell migration and invasion, as indicated by the wound-healing and transwell matrix invasion assays (Fig. 7b, c). These results indicate that TGF-β signaling activation is a critical mediator for IQGAP3-induced metastasis in HCC.

## Discussion

Invasion and metastasis are mainly responsible for a vast majority of cancer-associated deaths, including from HCC, wherein EMT plays a vital role in the invasion and metastasis of cancer cells. This study is the first to prove that IQGAP3 is dramatically elevated in HCC cells and tissues, and high expression of IQGAP3 in HCC is correlated with aggressive clinicopathological features. Furthermore, we found that IQGAP3 expression is an independent poor prognostic factor for overall survival, and its upregulation markedly enhances migration, invasion, and EMT in HCC cells in vitro and promotes metastasis of orthotopic hepatic tumors in nude mice. In contrast, silencing of *IQGAP3* inhibited invasion, EMT, and metastasis in vitro and in vivo. Our results demonstrate that IQGAP3 promotes invasion, EMT, and metastasis by activating the TGF-β/Smad signaling pathway. Importantly, the stimulatory effects of IQGAP3 on invasion and metastasis were attenuated by the TGF-β signaling inhibitor SB431542, indicating that TGF-β signaling is essential for IQGAP3-mediated pro-metastasis in HCC. Our findings provide new insights into the mechanisms of IQGAP3 that regulate invasion and metastasis in HCC.

Previous studies have indicated that IQGAP3 plays an important role in cell proliferation, adhesion, migration, and metastasis in various cancers [17, 18, 23, 24]. Furthermore, IQGAP3 has been identified as a multifunctional scaffold protein involved in cell adhesion and cell migration via interaction with diverse proteins [25]. As a scaffold protein, it is possible that IQGAP3 plays a critical role in the invasion and metastasis of cancer cells. For example, enforced expression of IQGAP3 accelerated the migration and invasion of lung cancer cells by interacting with ERK1 and promoting EGF-induced activation of ERK [17]. Moreover, another study reported that high expression of IQGAP1 and IQGAP3 was essential for development of invasive epidermal squamous cell carcinoma [24]. Notably, IQGAP3 levels are reportedly elevated in HCC [17, 19, 26]. However, the specific biological role of IQGAP3 remains largely unknown. In this study, we demonstrated that overexpression of IQGAP3 increases the invasion and metastasis abilities of HCC cells. The pro-invasion and metastasis roles of IQGAP3 in HCC were effectively inhibited by *IQGAP3* knockdown. Therefore, IQGAP3 could serve as a potential target for the development of novel anti-metastasis interventions in HCC.

Several lines of evidence indicate that the TGF-β signaling pathway plays crucial roles in regulating malignancy initiation, progression, and metastasis in several human cancers including mammary carcinoma, pancreatic cancer, colon carcinoma, and hepatocellular carcinoma [27]. Many studies have shown that inhibitors targeting TGF-β signaling significantly suppress tumor invasiveness and metastasis [28–31]. In the TGF-β signaling pathway, TGF-β1 receptor kinases phosphorylate Smad2 and Smad3 in the C-terminal residue, which further forms a complex with Smad4 and promotes nuclear translocation of the complex to regulate downstream gene expression, resulting in stimulation of EMT [32–35]. Our current findings illustrate that upregulated IQGAP3 expression promotes TGF-β1 expression in HCC cells, and TGF-β1 activation results in Smad2 and Smad3 phosphorylation, leading to upregulation of multiple downstream genes including *TGFB1*, *MMP2*, *LTBP1*, and *THBS1*. In the nude mice model, western blot showed that IQGAP3 overexpression was significantly correlated with high Snail, Twist1, MMP2, and MMP9 expression. Therefore, our findings confirm that IQGAP3 activates TGF-β–induced activity of Smad2 and Smad3 and transcriptional responses, which contribute to the malignant behavior of HCC. Thus, blockage of TGF-β/Smad signaling pathways by silencing *IQGAP3* may be a candidate targeted therapy for HCC cell metastasis.

TGF-β promotes the metastasis of cancer by inducing EMT, which is an important step in TGF-β–induced cancer cell migration and invasion [36, 37]. EMT is known to play an important role in migration, invasiveness, metastasis, and chemoresistance and has been highlighted as a potential therapeutic target in HCC [38, 39]. The EMT process carries cancerous cells away from the primary tumor, allowing them to invade the surrounding stromal tissue and propagate to distant organs [40]. This process involves downregulation of epithelial markers such as cytokeratin and adherens proteins like E-cadherin and upregulation of mesenchymal markers such as vimentin, fibronectin, and N-cadherin [41]. E-cadherin is the key components of the adherens junctions of cell membrane mediating cell-cell adhesion and cytoskeleton [42]. Herein, we demonstrated that IQGAP3 induces EMT in HCC cells, leading to downregulation of epithelial markers (E-cadherin) and upregulation of mesenchymal markers (vimentin, fibronectin, and N-cadherin).

## Conclusions

In conclusion, our study demonstrates that a high IQGAP3 expression level is closely correlated with poor overall survival of HCC patients. Importantly, we found that IQGAP3 promotes the invasion, EMT, metastasis of HCC cells in vitro and in vivo by activating TGF-β signaling. Together, these findings indicate that IQGAP3 functions as an important regulator of metastasis and EMT by constitutively activating the TGF-β signaling pathway in HCC. Determination of the precise roles of IQGAP3 in the pathogenesis and progression of HCC and activation of the TGF-β signaling pathway will help improve our understanding of the biological basis of cancer. Furthermore, therapy targeting IQGAP3 may facilitate the development of novel anti-metastasis strategies against HCC.

## Abbreviations

HCC: human hepatocellular carcinoma
EMT: epithelial to mesenchymal transition
TGF-β: transforming growth factor-β
IQGAP3: IQ motif-containing GTPase activating protein 3
qPCR: quantitative polymerase chain reaction
MMP2: matrix metalloproteinase-2
THBS1: thrombospondin 1
LTBP1: latent transforming growth factor beta binding protein-1
GAPDH: glyceraldehyde 3-phosphate dehydrogenase
GSEA: Gene Set Enrichment Analysis
TCGA: The Cancer Genome Atlas
IHC: immunohistochemistry
